# Supplementary material for: Influence of enclosure design on the behaviour and welfare of Pogona vitticeps
Source: PLoS One. 2025 Jun 5;20(6):e0322682. doi: 10.1371/journal.pone.0322682 (PMC12140227; doi:10.1371/journal.pone.0322682)
Supplement: S1 Appendix — (DOCX) [file pone.0322682.s001.docx]

**S1. Influence of the blind spot**

After all videos were scored, it became apparent that some lizards spent a large amount of time in the camera’s blind spot. Consequently, the percent of the day that a lizard was scored performing certain behaviours might not accurately reflect their actual behaviour. However, the camera’s blind spot was relatively small; the blind spot was the area directly below the camera (Fig S1.).


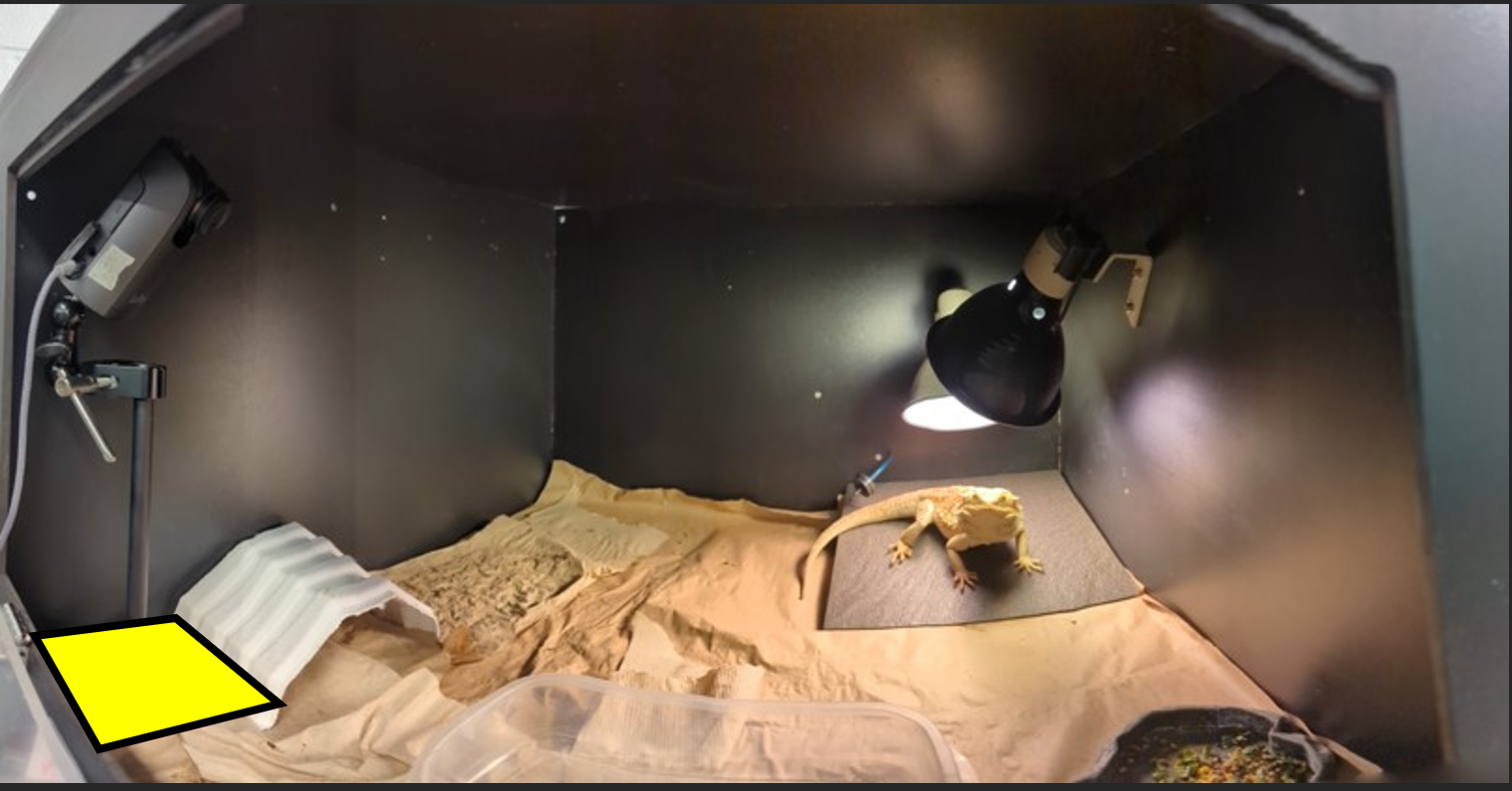

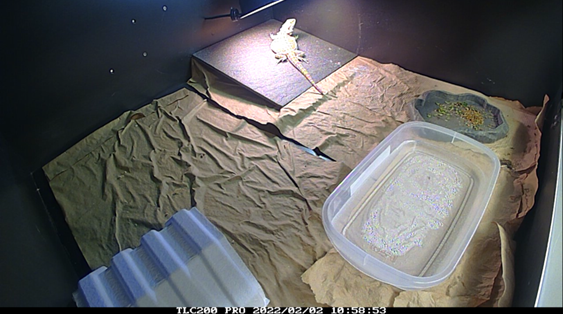


**B**

**A**

**Fig S1. Visualization of blind spot in video recordings of *Pogona vitticeps* behaviour.** (A) The camera’s position in the enclosure; the approximate area of the blind spot is highlighted with a yellow square. The area was approximately the size or smaller than the size of the stand’s base, which was 12.5cm^2^, rendering the blind spot less than 5% of the enclosure’s floor. (B) A screen shot from video play-back to highlighting how much area was hidden to the observer scoring behaviour.

Therefore, to remain completely within the blind spot for a long, interrupted period, a lizard would have to curl around the base of the camera or otherwise wedge themselves into a corner and not move. However, some lizards accumulated a large amount of time in the blind spot over several short bouts; for example, this might occur if the lizard was interacting with barriers and slid into the blind spot a number of times during this behaviour, or if the lizard perched atop their hide for several times. The way lizards accumulated time in the blind spot was inconsistent, but time spent in the blind spot was sometimes substantial (Fig S2). This prevented the assumption that a lizard in the blind spot was always inactive.


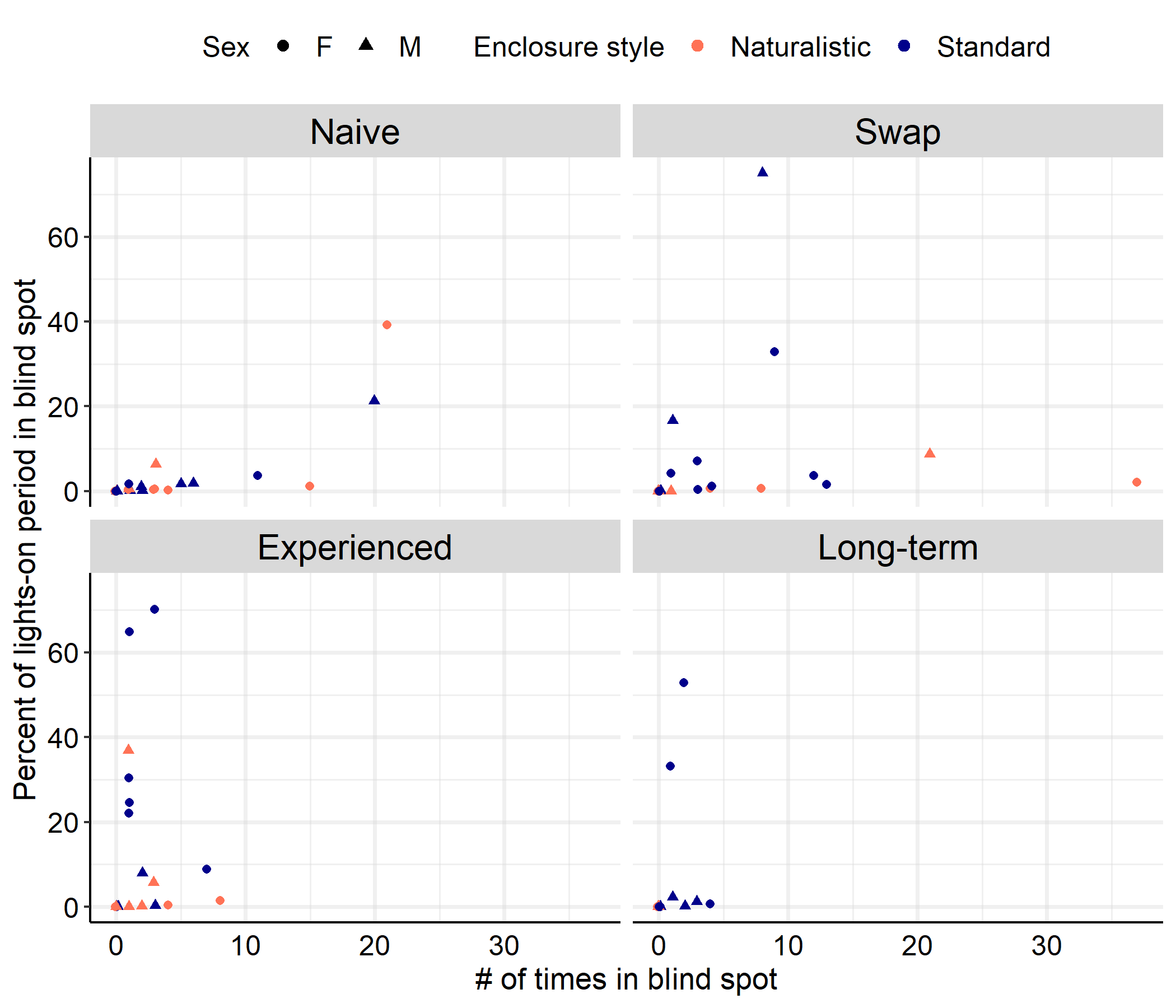


**Fig S2. Percent of time *Pogona vitticeps* spent within the blind spot depending on the number of entries.** Data are divided into the 4 time periods discussed in main body of text. At each time point, 24 lizards were observed, and each point represents a value for each lizard. Points are shaped and coloured based on sex and enclosure style, respectively. Although most lizards rarely entered the blind spot, some entered it frequently.

To account for the time spent in the blind spot, the percent of the day a lizard’s behaviour was performed was modified. Originally, the percent the lizard spent performing a behaviour was calculated by dividing the total amount of time they spent performing that behaviour with the amount of time the lizard’s lights were on. When modified, the percent the lizard spent performing a behaviour was calculated by dividing the total amount of time they spent performing that behaviour with the amount of time the lizard’s lights were on *subtracted by* any time spent in the blind spot. For more details on how this was done and how data were influenced by this method, see data analysis (available as a .html file in the dataset on Borealis: <https://borealisdata.ca/dataset.xhtml?persistentId=doi:10.5683/SP3/GIUPVO>).
