# Supplementary material for: Influence of enclosure design on the behaviour and welfare of Pogona vitticeps
Source: PLoS One. 2025 Jun 5;20(6):e0322682. doi: 10.1371/journal.pone.0322682 (PMC12140227; doi:10.1371/journal.pone.0322682)
Supplement: S2 Appendix — (ZIP) [file pone.0322682.s002.zip › SupportingInformation2.html]

Supporting information 2 for Influence of enclosure design on the behaviour and welfare of Pogona vitticeps


# Supporting information 2 for Influence of enclosure design on the behaviour and welfare of *Pogona vitticeps*

#### Melanie Denomme Stauder

#### 25 March, 2025

# 1 About

This file contains additional details about the analysis of
heterophil to lymphocyte ratios discussed in the manuscript titled
“Influence of enclosure design on the behaviour and welfare of
*Pogona vitticeps*”.

# 2 Packages & functions

```
library(dplyr)
library(ggplot2)
library(ggfortify)
library(ggrepel)
library(lme4)
library(lmerTest)
library(effects)
library(tidyr)
library(ggpubr)
library(nlme)
library(performance) 
library(gamlss)
library(easystats)
library(see)
library(car)
library(MuMIn)
library(AICcmodavg)
library(kableExtra)
library(gt)
library(insight)
library(superb)
library(png)
library(cowplot)
library(magick)
library(ggbeeswarm)
library(DHARMa)
library(sjPlot)
library(MASS)
library(emmeans)
library(marginaleffects)
library(epiDisplay)
library(Hmisc)
```

```
# This makes checking gamlss functions easier
checkgamlss <- function(x, ...) {
  normplot <- plot(x, main = "Fixed effects")
  autocorplot <- plot(x, ts = TRUE, main = "Fixed effects")
  wormplot <- wp(x)
  rsqr <- Rsq(x)
  #plots <- list(normplot, autocorplot, wp, rsqr)
}


# Reduces opacity of panel grid lines for future plots
col_grid <- rgb(235, 235, 235, 100, maxColorValue = 280)
```

# 3 Read in data

Read in data set that provides information about the white blood
cells sampled in lizards between April and June in 2024.

Load them in now and make sure the data are correctly labelled.

```
# For H:L ratios
hl <- read.csv("HLRatiosData.csv")

# Ensure data is in the proper format
hl$LizardNumber <- as.factor(hl$LizardNumber)
hl$LizardName <- as.factor(hl$LizardName)
hl$Sex <- as.factor(hl$Sex)
hl$CageType <- as.factor(hl$CageType)
hl$Slide <- as.factor(hl$Slide)
hl$DateScored <- as.POSIXct(hl$DateScored, format = "%Y-%m-%d")
hl$Lymphocytes <- as.integer(hl$Lymphocytes)
hl$Heterophils <- as.integer(hl$Heterophils)
hl$Monocytes <- as.integer(hl$Monocytes)
hl$OtherWBCs <- as.integer(hl$OtherWBCs)
hl$HLRatio <- as.numeric(hl$HLRatio)
hl$Used <- as.factor(hl$Used)
hl$DidResample <- as.factor(hl$DidResample)
```

# 4 H:L Ratios

In the spring of 2024, we took blood samples from all the dragons and
prepared blood smears with Wright-Geisma stain to examine the ratio of
heterophils to lymphocytes. Following other published methods, we
counted and identified the first 100 white blood cells, not including
thrombocytes. Sampling and initial analyses were also done while blind
to enclosure style and sex (i.e., blind to individual).

In other vertebrates, H:L ratios have been used as measures of
chronic stress (Davis et al.,
2008). This is because a chronically or often activated HPA axis can
damage the immune system, causing the amount of heterophils to increase
or the amount of lymphocytes to decrease. In a healthy animal,
lymphocytes should greatly outnumber heterophils.

Therefore, we would expect that lizards experiencing chronic stress
would have **higher** H:L ratios than those not
experiencing chronic stress. In our case, we expect the lizards in
standard enclosures to be chronically stressed compared to lizards in
naturalistic enclosures.

While collecting these data, we occasionally had to re-sample certain
individuals due to issues with slide quality. Because not all
individuals were re-sampled the same number of times, the analysis of
H:L ratios between lizards will use just 1 measurement for each
individual; for more details about this, see the final section of this
file which analyzes all the measurements taken.

**Note that, for health reasons, 1 lizard had their enclosure
style changed in April of 2024. They will be dropped from the following
analysis.**

```
hl2 <- hl %>%
  filter(LizardName != "fr.15") %>%
  droplevels()
```

## 4.1 Preliminary plots

```
hist(hl2$HLRatio)
```

```
hl2 %>%
  ggplot(aes(x = HLRatio, y = 1:nrow(hl2),
             colour = CageType, shape = Sex))+
  geom_point()+
  xlab("Range of the data") +
  ggtitle("Visual check for outliers")
```

## 4.2 Likelihood ratio tests

Use a likelihood ratio model to determine whether or not an
interaction should be included in the final model analyzed.

```
int.m <- glm(HLRatio ~ CageType*Sex,
                    family = Gamma(link = "log"),
                    data = hl2)
plot(simulateResiduals(fittedModel = int.m))
```

```
ad.m <- glm(HLRatio ~ CageType + Sex,
                    family = Gamma(link = "log"),
                    data = hl2)
plot(simulateResiduals(fittedModel = ad.m))
```

The additive model actually doesn’t fit the data that well.

However, it seems that the estimates can more or less be
predicted:

```
summary(ad.m)
```

```
## 
## Call:
## glm(formula = HLRatio ~ CageType + Sex, family = Gamma(link = "log"), 
##     data = hl2)
## 
## Coefficients:
##             Estimate Std. Error t value Pr(>|t|)    
## (Intercept)  -1.6210     0.2610  -6.211 4.57e-06 ***
## CageTypeSD    0.1870     0.2824   0.662    0.515    
## SexM         -0.2629     0.2846  -0.924    0.367    
## ---
## Signif. codes:  0 '***' 0.001 '**' 0.01 '*' 0.05 '.' 0.1 ' ' 1
## 
## (Dispersion parameter for Gamma family taken to be 0.3884072)
## 
##     Null deviance: 7.7598  on 22  degrees of freedom
## Residual deviance: 6.8424  on 20  degrees of freedom
## AIC: -35.656
## 
## Number of Fisher Scoring iterations: 8
```

Therefore, use the Anova function to determine if including the
interaction is significant.

```
car::Anova(int.m)
```

```
## Analysis of Deviance Table (Type II tests)
## 
## Response: HLRatio
##              LR Chisq Df Pr(>Chisq)  
## CageType       0.4862  1    0.48561  
## Sex            0.9509  1    0.32949  
## CageType:Sex   6.4923  1    0.01083 *
## ---
## Signif. codes:  0 '***' 0.001 '**' 0.01 '*' 0.05 '.' 0.1 ' ' 1
```

This is significant; dropping the interaction influences model
fit.

But because we know that the additive model doesn’t fit great, let’s
also compare the interaction-only model to models which include just
enclosure style or just sex.

```
cg.m <- glm(HLRatio ~ CageType,
                    family = Gamma(link = "log"),
                    data = hl2)
plot(simulateResiduals(fittedModel = cg.m))
```

```
sx.m <- glm(HLRatio ~ Sex,
                    family = Gamma(link = "log"),
                    data = hl2)
plot(simulateResiduals(fittedModel = sx.m))
```

```
lrtest(cg.m, int.m)
```

```
## Likelihood ratio test for MLE method 
## Chi-squared 2 d.f. =  7.529036 , P value =  0.02317878
```

```
lrtest(sx.m, int.m)
```

```
## Likelihood ratio test for MLE method 
## Chi-squared 2 d.f. =  7.119558 , P value =  0.02844511
```

```
car::Anova(int.m)
```

```
## Analysis of Deviance Table (Type II tests)
## 
## Response: HLRatio
##              LR Chisq Df Pr(>Chisq)  
## CageType       0.4862  1    0.48561  
## Sex            0.9509  1    0.32949  
## CageType:Sex   6.4923  1    0.01083 *
## ---
## Signif. codes:  0 '***' 0.001 '**' 0.01 '*' 0.05 '.' 0.1 ' ' 1
```

Both likelihood ratio tests are significant, therefore dropping the
interaction always seems to have an influence on the model fit.

Indeed, the Anova output demonstrates that dropping the interaction
seems to be the only thing that can influence model fit.

Now we know that we have to analyze results from a model which
includes an interaction between enclosure style and sex.

#### Model output

```
hl.cg.sx.glm <- glm(HLRatio ~ CageType*Sex,
                    family = Gamma(link = "log"),
                    data = hl2)

# Conclusions
summary(hl.cg.sx.glm)
```

```
## 
## Call:
## glm(formula = HLRatio ~ CageType * Sex, family = Gamma(link = "log"), 
##     data = hl2)
## 
## Coefficients:
##                 Estimate Std. Error t value Pr(>|t|)    
## (Intercept)      -2.0049     0.2531  -7.921 1.94e-07 ***
## CageTypeSD        0.7034     0.3042   2.312   0.0321 *  
## SexM              0.2883     0.3173   0.909   0.3749    
## CageTypeSD:SexM  -1.2372     0.4632  -2.671   0.0151 *  
## ---
## Signif. codes:  0 '***' 0.001 '**' 0.01 '*' 0.05 '.' 0.1 ' ' 1
## 
## (Dispersion parameter for Gamma family taken to be 0.2562908)
## 
##     Null deviance: 7.7598  on 22  degrees of freedom
## Residual deviance: 5.1785  on 19  degrees of freedom
## AIC: -40.339
## 
## Number of Fisher Scoring iterations: 5
```

```
r2(hl.cg.sx.glm)
```

```
## # R2 for Generalized Linear Regression
##   Nagelkerke's R2: 0.371
```

```
plot(allEffects(hl.cg.sx.glm))
```

```
# For p-values and model estimates
# Within an enclosure style, between sexes
emmeans(hl.cg.sx.glm, specs = pairwise ~Sex|CageType, adjust = "bonferroni")
```

```
## $emmeans
## CageType = EE:
##  Sex emmean    SE df lower.CL upper.CL
##  F    -2.00 0.253 19    -2.53   -1.475
##  M    -1.72 0.191 19    -2.12   -1.316
## 
## CageType = SD:
##  Sex emmean    SE df lower.CL upper.CL
##  F    -1.30 0.169 19    -1.65   -0.948
##  M    -2.25 0.292 19    -2.86   -1.639
## 
## Results are given on the log (not the response) scale. 
## Confidence level used: 0.95 
## 
## $contrasts
## CageType = EE:
##  contrast estimate    SE df t.ratio p.value
##  F - M      -0.288 0.317 19  -0.909  0.3749
## 
## CageType = SD:
##  contrast estimate    SE df t.ratio p.value
##  F - M       0.949 0.338 19   2.811  0.0111
## 
## Results are given on the log (not the response) scale.
```

```
# Within a sex, between enclosure style
emmeans(hl.cg.sx.glm, specs = pairwise ~CageType|Sex, adjust = "bonferroni")
```

```
## $emmeans
## Sex = F:
##  CageType emmean    SE df lower.CL upper.CL
##  EE        -2.00 0.253 19    -2.53   -1.475
##  SD        -1.30 0.169 19    -1.65   -0.948
## 
## Sex = M:
##  CageType emmean    SE df lower.CL upper.CL
##  EE        -1.72 0.191 19    -2.12   -1.316
##  SD        -2.25 0.292 19    -2.86   -1.639
## 
## Results are given on the log (not the response) scale. 
## Confidence level used: 0.95 
## 
## $contrasts
## Sex = F:
##  contrast estimate    SE df t.ratio p.value
##  EE - SD    -0.703 0.304 19  -2.312  0.0321
## 
## Sex = M:
##  contrast estimate    SE df t.ratio p.value
##  EE - SD     0.534 0.349 19   1.528  0.1430
## 
## Results are given on the log (not the response) scale.
```

```
# Values
hl2 %>%
  group_by(Sex, CageType) %>%
  summarise(
    n = length(HLRatio),
    med = median(HLRatio, na.rm = TRUE),
    ci.low = parameters::ci(HLRatio)$CI_low,
    ci.high = parameters::ci(HLRatio)$CI_high,
    ci.width = (ci.high - ci.low))
```

```
## # A tibble: 4 × 7
## # Groups:   Sex [2]
##   Sex   CageType     n    med ci.low ci.high ci.width
##   <fct> <fct>    <int>  <dbl>  <dbl>   <dbl>    <dbl>
## 1 F     EE           4 0.133  0.104    0.168   0.0643
## 2 F     SD           9 0.247  0.106    0.519   0.413 
## 3 M     EE           7 0.125  0.0506   0.369   0.319 
## 4 M     SD           3 0.0952 0.0902   0.129   0.0390
```

```
# Count lizards in each enclosure style and sex
table(hl2$CageType, hl2$Sex)
```

```
##     
##      F M
##   EE 4 7
##   SD 9 3
```

Interestingly, we find that the **interaction of sex and
enclosure style** is influential.

### 4.2.1 Final plot

# 5 Additional analyses

## 5.1 H:L analysis with means

We experienced a lot of issues with preparing slides for the
measurement of H:L ratios. As a result, we measured H:L ratios for half
of the lizards more than once. In the analysis, however, we only use the
measurement that was the most recent or had the fewest recorded issues.
To make sure this did not bias my measurements, we will check to see if
the analysis with all the data comes to any different conclusions.

Try calculating the mean and filter out repeated data points.

```
# Drop lizard who was moved into their enclosure style more recently
hl.all <- hl.all %>% filter(LizardName != "fr.15") %>% droplevels()

hl.all <- hl.all %>%
  group_by(LizardName) %>%
  mutate(meanHLRatio = mean(HLRatio))

# Count # times resampled
hl.all <- hl.all %>%
  group_by(LizardName) %>%
  mutate(TimesResampled = length(DidResample == "Y")) 

hl.all$TimesResampled <- as.factor(hl.all$TimesResampled)

hl.all.used <- hl.all %>%
  filter(Used == "Y") %>%
  droplevels()

hl.all.long <- pivot_longer(hl.all.used,
                           cols = c("HLRatio", "meanHLRatio"),
                           names_to = "Ratio",
                           values_to = "Value")
```

### 5.1.1 Checking means vs. selected values

Now plot to see how much the re-sampled individuals deviated from
their non-resampled counterparts AND see how much the used sample
deviated from the mean sample.

```
hl.all %>%
  ggplot(aes(x = DidResample, y = meanHLRatio, shape = DidResample, colour = DidResample)) +
  geom_boxplot()+
  geom_point()
```

```
hl.all.long %>%
  ggplot(aes(x = LizardName, y = Value)) +
  geom_point(aes(shape = Ratio, colour = TimesResampled))+
  geom_line(aes(group = LizardName, colour = TimesResampled)) +
  theme(axis.text.x = element_text(angle = 45,hjust = 1)) +
  scale_y_continuous(limits = c(0, 0.6), 
                     breaks = seq(0, 0.6, by = 0.05))+
  ylab("H:L ratio") +
  scale_colour_discrete(name = "# times sampled")+
  scale_shape_discrete(name = "",
                       labels = c("Value used for analysis",
                                  "Average value"))
```

```
hl.all.long %>%
  filter(DidResample == "Y") %>%
  ggplot(aes(x = LizardName, y = Value)) +
  geom_point(aes(shape = Ratio, colour = TimesResampled))+
  geom_line(aes(group = LizardName, colour = TimesResampled)) +
  theme(axis.text.x = element_text(angle = 45,hjust = 1)) +
  scale_y_continuous(limits = c(0, 0.6), 
                     breaks = seq(0, 0.6, by = 0.05)) +
  scale_shape_manual(values = c(8, 16)) +
  ylab("H:L ratio") +
  scale_colour_manual(name = "# times sampled",
                        values = c("firebrick", "darkgoldenrod",
                                   "chartreuse3"))+
  scale_shape_discrete(name = "",
                       labels = c("Value used for analysis",
                                  "Average value"))
```

There was some variability, but we can also see that the sample we
used (labelled “HLRatio”) was not consistently higher or lower than the
mean (labelled “meanHLRatio”).

Now let’s add in our factors of interest and re-do the analysis to
check how this influences things.

### 5.1.2 Repeat analyses

Now repeat the analysis.

```
int.m <- glm(meanHLRatio ~ CageType*Sex,
                    family = Gamma(link = "log"),
                    data = hl.all.used)
plot(simulateResiduals(fittedModel = int.m))
```

```
ad.m <- glm(meanHLRatio ~ CageType + Sex,
                    family = Gamma(link = "log"),
                    data = hl.all.used)
plot(simulateResiduals(fittedModel = ad.m))
```

```
lrtest(ad.m, int.m)
```

```
## Likelihood ratio test for MLE method 
## Chi-squared 1 d.f. =  3.991396 , P value =  0.04573315
```

Like last time, we find the interaction is significant.

```
hlmeans.cg.sx.glm <- glm(meanHLRatio ~ CageType*Sex,
                    family = Gamma(link = "log"),
                    data = hl.all.used)

# Conclusions
summary(hlmeans.cg.sx.glm)
```

```
## 
## Call:
## glm(formula = meanHLRatio ~ CageType * Sex, family = Gamma(link = "log"), 
##     data = hl.all.used)
## 
## Coefficients:
##                 Estimate Std. Error t value Pr(>|t|)    
## (Intercept)      -1.9632     0.2526  -7.773 2.57e-07 ***
## CageTypeSD        0.6350     0.3036   2.092   0.0501 .  
## SexM              0.2186     0.3166   0.690   0.4983    
## CageTypeSD:SexM  -0.9778     0.4622  -2.115   0.0478 *  
## ---
## Signif. codes:  0 '***' 0.001 '**' 0.01 '*' 0.05 '.' 0.1 ' ' 1
## 
## (Dispersion parameter for Gamma family taken to be 0.2551849)
## 
##     Null deviance: 7.8202  on 22  degrees of freedom
## Residual deviance: 5.8838  on 19  degrees of freedom
## AIC: -37.552
## 
## Number of Fisher Scoring iterations: 5
```

```
r2(hlmeans.cg.sx.glm)
```

```
## # R2 for Generalized Linear Regression
##   Nagelkerke's R2: 0.280
```

```
plot(allEffects(hlmeans.cg.sx.glm))
```

```
# For p-values and model estimates
# Within an enclosure style, between sexes
emmeans(hlmeans.cg.sx.glm, specs = pairwise ~Sex|CageType, adjust = "bonferroni")
```

```
## $emmeans
## CageType = EE:
##  Sex emmean    SE df lower.CL upper.CL
##  F    -1.96 0.253 19    -2.49   -1.435
##  M    -1.74 0.191 19    -2.14   -1.345
## 
## CageType = SD:
##  Sex emmean    SE df lower.CL upper.CL
##  F    -1.33 0.168 19    -1.68   -0.976
##  M    -2.09 0.292 19    -2.70   -1.477
## 
## Results are given on the log (not the response) scale. 
## Confidence level used: 0.95 
## 
## $contrasts
## CageType = EE:
##  contrast estimate    SE df t.ratio p.value
##  F - M      -0.219 0.317 19  -0.690  0.4983
## 
## CageType = SD:
##  contrast estimate    SE df t.ratio p.value
##  F - M       0.759 0.337 19   2.254  0.0362
## 
## Results are given on the log (not the response) scale.
```

```
# Within a sex, between enclosure style
 emmeans(hlmeans.cg.sx.glm, specs = pairwise ~CageType|Sex, adjust = "bonferroni")
```

```
## $emmeans
## Sex = F:
##  CageType emmean    SE df lower.CL upper.CL
##  EE        -1.96 0.253 19    -2.49   -1.435
##  SD        -1.33 0.168 19    -1.68   -0.976
## 
## Sex = M:
##  CageType emmean    SE df lower.CL upper.CL
##  EE        -1.74 0.191 19    -2.14   -1.345
##  SD        -2.09 0.292 19    -2.70   -1.477
## 
## Results are given on the log (not the response) scale. 
## Confidence level used: 0.95 
## 
## $contrasts
## Sex = F:
##  contrast estimate    SE df t.ratio p.value
##  EE - SD    -0.635 0.304 19  -2.092  0.0501
## 
## Sex = M:
##  contrast estimate    SE df t.ratio p.value
##  EE - SD     0.343 0.349 19   0.983  0.3378
## 
## Results are given on the log (not the response) scale.
```

This does render the difference for female lizards in naturalistic or
standard enclosures barely non-significant (p = 0.0501). However, we
still find that, for lizards in standard enclosures, female lizards had
higher HL ratios (p = 0.0362).

### 5.1.3 Plot

```
avgpred2 <- avg_predictions(hlmeans.cg.sx.glm,
                  type = "response",
                  #vcov = vcvrobust,
                  variables = c("Sex", "CageType"))

# Plot
ggplot() +
  geom_pointrange(data = avgpred2,
                  aes(x = Sex, y = estimate,
                      ymin = conf.low, ymax = conf.high, 
                      colour = CageType, group = CageType), 
                  position = position_dodge(width = 0.5),
                  size = 1, linewidth = 1.5) +
  geom_point(data = hl.all.used,
             aes(x = Sex, y = meanHLRatio, 
                 group = CageType, colour = CageType,
                 shape = DidResample),
              position = position_jitterdodge(dodge.width = 0.5,
                                             jitter.width = 0.3), 
             alpha = 0.6, size = 3) +
  theme(axis.line.y = element_line(color="black"),
        axis.title.y = element_text(colour="black",size = 15),
        axis.text.y = element_text(colour="black", size = 15),
        axis.line.x = element_line(colour = "black"),
        axis.text.x = element_text(colour="black", size = 15),
        axis.ticks.x = element_blank(),
        axis.title.x = element_text(colour="black",size = 15),
        #legend.position = c("right", "right"),
        legend.background = element_blank(),
        panel.grid = element_line(linetype = "solid", colour = col_grid),
        panel.grid.major.x = element_blank(),
        panel.background=element_rect(fill="white",colour="white")
        )+
  scale_colour_manual(name = "Enclosure style",
                      labels = c("Naturalistic", "Standard"),
                      values = c("coral1", "darkblue"))+
  scale_fill_manual(name = "Enclosure style",
                    labels = c("Naturalistic", "Standard"),
                      values = c("coral1", "darkblue")) +
  scale_shape_manual(name = "Resampled",
                     labels = c("No", "Yes"),
                     values = c(16, 17))+
  scale_x_discrete(labels = c("Female", "Male"))+
  scale_y_continuous(breaks = seq(from = 0, to = 0.6, by = 0.1))+
  xlab("Sex")+
  coord_cartesian(clip = "off") +
  ylab("H:L ratios") +
  # FEMALE SAMPLE SIZES
  annotate("text", x = 0.85, y = 0, label = "n = 4",
            color = "coral1", size = 5) +
  annotate("text", x = 1.16, y = 0, label = "n = 9", 
           colour = "darkblue", size = 5) +
  # MALE SAMPLE SIZES
  annotate("text", x = 1.85, y = 0, label = "n = 7",
            color = "coral1", size = 5) +
  annotate("text", x = 2.16, y = 0, label = "n = 3", 
           colour = "darkblue", size = 5) +
  # SIGNIFICANCE
  # Naturalistic vs. Standard: Females
  showSignificance(x = c(0.87, 1.13), y = 0.40, 
                   width = c(-0.15,-0.02),
                   "p = 0.0501",
                   segmentParams = list(
                     linewidth = 0.9, colour = "black"),
                   textParams = list(
                     size = 4, colour = "black"))+
  # Female vs. Male: Standard enclosures
  showSignificance(x = c(1.12, 2.12), y = 0.45, 
                   width = c(-0.01, -0.25),
                   "p = 0.036",
                   segmentParams = list(
                     linewidth = 0.9, colour = "darkblue"),
                   textParams = list(
                     size = 4, colour = "darkblue"))
```

Interestingly, re-sampling didn’t seem to change any of the extremes.
Compared to the original graph, the extreme values of standard-housed
females are almost the same. In fact, the lowest value for
standard-housed females is a little higher in the original analysis;
using the mean made this value lower. This seems to be part of the
reason why the p-value gets bigger.

There also appears to be slightly less variability in the mean values
compared to the original ones for naturalistic-housed females; in the
new figure, the values for these individuals are grouped more tightly.
As a result, the predicted confidence interval for naturalistic-housed
females is ever so slightly higher.

So, using the mean values shifts the range for standard-housed
females slightly lower, and shifts the range for naturalistic-housed
females slightly higher.

It also reduces the p-value of the between-sex effect partially by
increasing the variability of the data recorded for males in
standard-style enclosures; their highest value gets higher. The spread
of data for naturalistic-housed males remains essentially the same.
