# Supplementary material for: Influence of enclosure design on the behaviour and welfare of Pogona vitticeps
Source: PLoS One. 2025 Jun 5;20(6):e0322682. doi: 10.1371/journal.pone.0322682 (PMC12140227; doi:10.1371/journal.pone.0322682)
Supplement: Attachment — Submitted filename: Response to Reviewers.docx [file pone.0322682.s004.docx]

**General comments:**

Upon review, we realized that for the influence of sex on IWB at the swap point, the direction was incorrectly stated in the results (line 500) and correctly stated in the figure caption. We have now fixed this error.

Also, in this response document, we have occasionally copied text from the original manuscript to effectively highlight any edits we have made. However, as a result, any citations from this copied text are no longer in their original format; i.e., they are provided as names, rather than numbers.

**Responses to reviewers:**

Reviewer #1: Dear authors, I would like to congratulate you on your work, which is very interesting and relevant. I really enjoyed reading it. However, I have a few suggestions that could make the manuscript even better. In general, the points for improvement are:
1 - There is no information about the biology and behavior of Pogona in the introduction. I suggest inserting a paragraph about this. One idea would be to add the objectives of the study on line 86.

Thank you for this comment; including such detail would better highlight the rationale for several parts of the study. Therefore, we have added a brief mention of wild *P. vitticeps* behaviour on line 102 (new text underlined below):

Although *P. vitticeps* are largely inactive in the wild (Bernich et al., 2022; Wild et al., 2022), too little or too much time inactive could indicate that the enclosure is failing to accommodate their needs or behaviours in some way; for example, lizards may bask excessively if the temperatures provided are inadequate or perform repetitive behaviours incessantly in attempts to escape (see Denommé & Tattersall, 2025).

To keep the primary focus of the manuscript on assessing the behaviour and welfare of *P. vitticeps*, a full paragraph about the natural history of *P. vitticeps* has been added to the methods, as the first paragraph of the “Animals & husbandry” section.

2 - The differences between the two types of enclosure. For me, the so-called natural enclosure didn't differ much from the so-called standard one.

We chose to label the “naturalistic” enclosure as naturalistic for several reasons. Primarily, “naturalistic” best described the enclosure’s appearance compared to the standard enclosures and also captured the intended outcome of the modifications in the naturalistic enclosure (i.e., to simulate aspects of the natural environment and promote natural behaviours). Although the naturalistic enclosures may also be described as “complex”, this label would have shifted the focus away from the rationale and intended outcome of the furnishings in the naturalistic enclosures. Indeed, because naturalistic enclosures included only 2 *additional* furnishings (cork bark with paper hide underneath) but *modified* 2 others (substrate and the naturalistic hide in the front-left quadrant), the term “complex” may have caused readers to overlook these modifications and focus on the number of furnishings.

Furthermore, the contrast between the standard and naturalistic enclosures may effectively replicate the conditions provided to pet reptiles in captivity. Reptiles are often provided, at minimum, a hide and a warm/basking spot (representing the “standard” enclosure), but loose substrate and climbing apparatuses may be less common depending on the species (Cargill et al., 2022; D’Cruze et al., 2020; Howell et al., 2022; Howell & Bennett, 2017). Therefore, our enclosure designs were chosen to have the broadest potential impact, as the pet trade represents a large portion of the reptiles currently kept in captivity (although *P. vitticeps* are also becoming an increasingly popular model organism for certain research; Fenk et al., 2024). Both enclosure designs were also approved by our local Animal Care Committee and represent aspects that may be feasible in a research environment.

The label “naturalistic” was also chosen as the label “enriched” may have been misleading. In the literature, “enriched” will typically (and should exclusively; Olsson & Dahlborn, 2002) refer to modifications that *improve* the animal’s welfare in some way, shape, or form (Ratuski & Weary, 2022). As we had no evidence that any of the resources provided to lizards improved the lizard’s well-being beyond the basic requirements, the label “enriched” was avoided. This is also why, throughout the article, we referred to the items in the enclosures as “resources” or “furnishings” rather than “enrichments” (a similar convention has been used in other literature; e.g., Cait et al., 2024).

Therefore, although we agree with the reviewer that the naturalistic enclosures did not closely replicate the lizard’s natural environment, these enclosures can still be viewed as “naturalistic” when compared to the standard enclosures. And, as this manuscript focused only on comparisons between these two enclosure styles, the label “naturalistic” was arguably the most accurate in the context of our experiment. Regardless, to make it clearer that we did not intend for “naturalistic” enclosures to fully replicate the natural environment, we have modified the following sentence in the introduction (new text underlined below):

Because naturalistic enclosures will include additional furnishings and attempt to mimic aspects of the natural environment, we expect these enclosures will better facilitate a lizard’s agency and that the welfare of lizards in these enclosures will be better than those in standard enclosures; we also expect this influence to be evident regardless of the order in which enclosure styles are experienced, and that an enclosure style’s influence will increase the longer it is inhabited.

**References cited in response**:

Cait, J., Winder, C. B., & Mason, G. J. (2024). How much “enrichment” is enough for laboratory rodents? A systematic review and meta-analysis re-assessing the impact of well-resourced cages on morbidity and mortality. *Applied Animal Behaviour Science*, *278*, 106361. <https://doi.org/10.1016/j.applanim.2024.106361>

Cargill, B., Benato, L., & Rooney, N. (2022). A survey exploring the impact of housing and husbandry on pet snake welfare. *Animal Welfare*, *31*(2), 193–208. <https://doi.org/10.7120/09627286.31.2.004>

D’Cruze, N., Paterson, S., Green, J., Megson, D., Warwick, C., Coulthard, E., Norrey, J., Auliya, M., & Carder, G. (2020). Dropping the Ball? The Welfare of Ball Pythons Traded in the EU and North America. *Animals*, *10*(3), 413. <https://doi.org/10.3390/ani10030413>

Howell, T. J., & Bennett, P. C. (2017). Despite their best efforts, pet lizard owners in Victoria, Australia, are not fully compliant with lizard care guidelines and may not meet all lizard welfare needs. *Journal of Veterinary Behavior*, *21*, 26–37. <https://doi.org/10.1016/j.jveb.2017.07.005>

Howell, T. J., Warwick, C., & Bennett, P. (2022). Pet management practices of frog and turtle owners in Victoria, Australia. *Veterinary Record*, *191*(12), e2180. <https://doi.org/10.1002/vetr.2180>

Olsson, I. A. S., & Dahlborn, K. (2002). Improving housing conditions for laboratory mice: A review of “environmental enrichment.” *Laboratory Animals*, *36*(3), 243–270. <https://doi.org/10.1258/002367702320162379>

Ratuski, A. S., & Weary, D. M. (2022). Environmental Enrichment for Rats and Mice Housed in Laboratories: A Metareview. *Animals*, *12*(4), Article 4. <https://doi.org/10.3390/ani12040414>

3 - Where was the study that evaluated the inactivity of pogona in the wild carried out? Was it a review? Was it an experiment? Should this study be considered the standard for the species? In the discussion you put some sentences about how pogonas are not very active in the wild. I think this should come up in the discussion as a possible point to take into account when looking at the results.
In this manuscript, we cite several different articles to support the general inactivity of *Pogona* in the wild: (Bernich et al., 2022; Thompson & Thompson, 2003; Wotherspoon, 2007). Upon review, we realized we failed to cite one important paper, however; (Wild et al., 2022). This paper has now been included wherever we have stated that *P. vitticeps* are largely inactive in the wild.

This research is all primary and, collectively, examined the behaviour of 230 *P. vitticeps*, 19 *P. minor*, and 22 *P. barbata* in the wild. *P. minor* and *P. barbata* were tracked either by using a spool-and-thread technique (Thompson & Thompson, 2003; Wotherspoon, 2007) and *P. vitticeps* were tracked using radiotelemetry and GPS tracking (Bernich et al., 2022; Wild et al., 2022). Consequently, the method each article used to estimate activity or movement rates differed. As we are unfamiliar with the techniques used to estimate activity levels in the wild, we are unable to comment on what the standard method should be; however, the relatively consistent estimates of inactivity produced by these variable techniques arguably bolsters the reliability of each article’s findings. Furthermore, the relative inactivity of *P. vitticeps* in the wild was mentioned in our original discussion (see, e.g., lines 779-786, 791-792).

For these reasons, we have not elected to add new details to our manuscript.

**References cited in response**:

Bernich, A., Maute, K., Contador-Kelsall, I. C., Story, P. G., Hose, G. C., & French, K. (2022). Space use and daily movement patterns in an arid zone agamid lizard. *Wildlife Research*, *49*(6), 557–570. <https://doi.org/10.1071/WR20152>

Thompson, S. A., & Thompson, G. G. (2003). The western bearded dragon, *Pogona minor* (Squamata: Agamidae): An early lizard coloniser of rehabilitated areas. *Journal of the Royal Society of Western Australia*, *86*, 1–6.

Wild, K. H., Roe, J. H., Schwanz, L., Georges, A., & Sarre, S. D. (2022). Evolutionary stability inferred for a free ranging lizard with sex‐reversal. *Molecular Ecology*, *31*(8), 2281–2292. <https://doi.org/10.1111/mec.16404>

Wotherspoon, A. D. (2007). *Ecology and management of Eastern Bearded Dragon* Pogona barbata*.* [PhD Thesis]. University of Western Sydney.

Please find my comments and suggestions below:
Line 53: (validated, i.e. [10]). I think this needs to be rewritten to make the text clearer. Perhaps, instead of citing the reference, cite some of the validated methods in the sentence.

This is a great point; it would be judicious to make the process of validating indicators of animal welfare more commonly known. We have added a sentence in these brackets to briefly describe the process from Browning, 2023 (original reference #10).

New text underlined below:

However, little research exists about reptile behaviour compared to other vertebrates (Bonnet et al., 2002; Rosenthal et al., 2017), and there is a considerable lack of validated welfare indicators for reptiles (i.e., validated by repeated testing with multiple other indicators that should all relate to the same state of welfare; (Browning, 2023)), ultimately making it difficult to determine what husbandry practices may be adequate.

Lines 86-88: This sentence about gender could be moved to the methodology (suddenly in the data analysis section). But, if you prefer to keep it here in the introduction, my suggestion is that you put why you don't expect a different response from the sex in relation to the type of enclosure.

Thank you for highlighting this. As suggested, we have modified the mention of sex at this point and in the methods.

We have modified the fourth paragraph of the introduction in the following manner (new text underlined and any text that has been deleted is struck out):

However, because the timing and order by which animals are exposed to enrichment can influence its efficacy (e.g., (Kalliokoski et al., 2012; Nagabaskaran et al., 2022)), we will investigate each hypothesis at multiple points in time, before and after lizards have experienced both naturalistic and non-naturalistic conditions. ~~In addition, because sex can influence the outcomes that will be measured [37,38], it will be included during analysis, but we do not expect that enclosure style will differently influence lizards based on their sex.~~ Because naturalistic enclosures will include additional furnishings and attempt to mimic aspects of the natural environment, we expect these enclosures will better facilitate a lizard’s agency and that the welfare of lizards in these enclosures will be better than those in standard enclosures; we also expect this influence to be evident regardless of the order in which enclosure styles are experienced, and that an enclosure style’s influence will increase the longer it is inhabited. In addition, although sex is known to influence some of the outcomes that will be measured (Bernich et al., 2022; Howard & Jaensch, 2021), enclosure style should not influence lizards differently based on their sex; because we expect that naturalistic enclosures will better facilitate agency, the lizard’s behaviour should be accommodated and their welfare should be improved regardless of the potentially unique motivations of each sex.

We have also modified the “Data analysis” section, 3 paragraphs in to the “General methods for all analyses” section in the following manner (new text underlined):

Because sex could influence the outcomes measured (Bernich et al., 2022; Howard & Jaensch, 2021), it was included during analysis; however, we did not have *a priori* reasons to expect that sex would influence the potential effects of enclosure style. Likelihood ratio tests were used to identify which factors (e.g., enclosure style, sex, etc.) in what combination (e.g., interaction, additive, separate) most influenced model fits and thereby identify which models would be further investigated. First, likelihood…

Lines 98-99: cite references to Pogona's natural behavior.

Please see our response to the first comment where we have now provided additional information on the biology of *Pogona*.

Lines 105-108: Here I think it depends. For example, if inactivity drops significantly while the diversity of positive behaviors increases? Would this change in the level of inactivity be considered bad? Probably not.

It is often difficult to relate the amount of time spent inactive to a definitive affective state, whether positive or negative. For this reason, we argued that **large deviations** from what wild lizards perform could highlight some issue, implying that spending an *extreme* amount of time inactive is the issue – whether this means that the lizard spends very little or a significant amount of time inactive. However, because it would be erroneous to assume that we know how long *P. vitticeps* “should” spend inactive each day, we aimed to use the behaviour of wild *P. vitticeps* to provide context to our lizard’s behaviours; the reasons for this decision are discussed in the manuscript.

The situation the reviewer has described (where inactivity decreases because the animal is performing more beneficial behaviours) does not refute this argument; in this situation, inactivity has strayed *away* from an extreme to a more moderate level, as the animal is performing a greater variety of behaviours. Furthermore, because lizards are ectothermic and must spend at least some portion of their day inactive to bask, it is reasonable to argue that spending very little time inactive is also problematic – this would mean the animal is not effectively thermoregulating, possibly because they are highly motivated to perform stress-related behaviours.

To make our reasoning clearer, we have added slightly more detail for explanation on lines 105-108 (new text underlined below):

“In either case, large deviations (i.e., extreme values) in the amount of time inactive relative to wild *P. vitticeps* may reflect that the behaviours that captive lizards can perform are restricted and, as a consequence, so too would their agency be restricted (Veasey et al., 1996).”

Lines 108-113: Likewise here, it depends. Exacerbated use of an area can be related to what resource that area provides, and that's not necessarily a bad thing. In fact, you found this in your work. The most used area was the lamp area and the structure where the animals could climb. So I think these statements should be more lenient.

Indeed, we do not disagree with this statement; animals may occupy some areas of an enclosure far more than others, and that could reflect that they are using the enclosure properly and their agency is not restricted. We would not expect enclosure use to be even across all areas. This was why our argument was that “…lizards in naturalistic enclosures should have either **similar or less skewed enclosure use patterns** **compared to those in standard enclosures**…” Therefore, by comparing enclosure use between the two enclosure types – and not to an arbitrary expectation of what enclosure use “should” be – we aimed to determine if some areas of the enclosure were not being used effectively.

In other words, if the use of an area in the enclosure increased when cork bark was added, this could indicate that the area benefits by the addition of cork bark – before this addition, the area was “avoided”. In this case, the lizards with cork bark would have *less skewed* enclosure use patterns than those in standard cages, because the addition of cork bark “evened” out their enclosure use patterns, and their agency would be facilitated by cork bark.

However, if the addition of cork bark caused an area to be used less, this could indicate that the cork bark was aversive, and therefore the cork bark had a negative effect on the lizard’s agency. In this case, lizards with cork bark would have *more skewed* enclosure use patterns, as they avoided one area of the enclosure more than those without cork bark.

Finally, if adding cork bark had no impact on enclosure use – i.e., lizards in both enclosure styles had a *similar pattern* of enclosure use – this would mean that the cork bark had, at the very least, no negative influence on the lizard’s agency. This aimed to account for situations where a barren area could facilitate the lizard’s agency, but cork bark *could also* facilitate agency; i.e., situations where lizards in *both* enclosure styles had their agency effectively facilitated. This could occur for a number of reasons that we may not be able to anticipate or reasons that are outside of our control (e.g., personality, sex, season, time of day, life stage, etc.). Indeed, because it is not feasible to know what “optimal” enclosure use looks like (as we cannot know each lizard’s motivations), we compared enclosure use between the two enclosure styles; in doing so, we could demonstrate that naturalistic enclosures at least had no *negative* impact on the lizard’s agency.

To make our argument clearer, we have modified the sentence in question (new text underlined below):

“When compared between enclosure styles, areas that are avoided or over-used could indicate that lizards perceive these areas (or the furnishings they include) as aversive, inadequate, or useless.

Line 118: But wouldn't inactivity be used as a measure of low well-being or of the enclosure's failure to accommodate the lizards' needs? After seeing the results, I'd modify it here and say that you took two measures of inactivity (you explain this later in the paper, because ILS was used in a particular context due to the low number of records).

Inactive with leg stretched (ILS) was a single behaviour that was used as an indicator of positive welfare for the reasons described on lines 120-130 (i.e. our second study objective). Because ILS may indicate positive welfare, if naturalistic enclosures improve the lizard’s welfare, they should be more “relaxed” and therefore be performing more ILS. Because the relationship between inactivity and welfare is complex (as discussed in earlier comments), ILS was kept distinct from the earlier discussion of inactivity in the manuscript.

The first measurement of inactivity described was used to examine if the behaviour of lizards in naturalistic enclosures was better accommodated (i.e. our first study objective), whereas ILS was used to examine if the welfare of lizards in naturalistic enclosures was better. Though related – because an animal whose behaviour is effectively accommodated would likely have good welfare – these hypotheses are distinct. Indeed, it was possible for us to have found that both enclosure styles effectively accommodated the lizard’s behaviours, but only one enclosure style improved welfare (or vice versa). This could occur for a number of reasons; for example, one enclosure style could have improved the lizard’s ability to cope with stressors, resulting in lower overall stress but no difference in the “regular” behavioural patterns. This is why the predictions supporting each hypothesis are described in separate paragraphs and not numbered consecutively.

To make it clearer that these were distinct objectives, we have modified the beginning of the paragraph which introduces ILS (new text underlined below):

In addition to assessing if naturalistic enclosures better accommodate a lizard’s behaviour, the welfare of lizards in naturalistic enclosures and standard enclosures will be investigated by comparing: (1) the amount of time spent inactive with legs stretched (ILS),

Line 186: Were the lizards captured and handled for this purpose? The answer is yes, as you'll find out later in the paper. From the results, do you think this might have had an influence on the parameters observed?

Yes, lizards were captured and handled for weekly health checks. Steps were taken to ensure this was not a particularly aversive event; lizards were transported from their enclosures in smaller transport containers and were always offered live insects before or during health checks. Lizards also should have been habituated to health checks; prior to the first observation period in June/July 2022, they had been handled at least once per week for 8 months.

Furthermore, except for one lizard in January 2023, behaviours were always recorded at least 12 hours after handling. The exception to this was a lizard who was taken out of their cage for ~2 minutes to receive a treatment for a minor scale issue on their beard; however, the lizard was not picked up, as they were removed and replaced from their enclosure while resting on cork bark. The lizard also experienced these treatments for approximately 50 days before this point.

For these reasons, we do not believe weekly health checks had an influence on the behaviours measured. Furthermore, even if health checks had influenced the lizard’s behaviours, this influence should not have been consistent. Health checks were almost always done on Fridays, and behavioural recordings were collected on random days throughout the week (except for the swap time point, where lizards were observed as soon as possible after their enclosure style had changed, but this is mentioned in our manuscript).

Lines 214-221: What behavioral data collection method was used? Focal Instantaneous with registers every 2 seconds?

Behaviours were measured using focal sampling (as there was only 1 lizard in the enclosure) and continuous recording; every behaviour the lizard performed was recorded throughout the 12-hour lights-on period. However, because timelapse cameras took 1 image every 2 seconds, this did limit the “resolution” of the behaviours we could observe. For example, if the lizard moved particularly fast or performed a behaviour that could begin and end within 2 seconds (e.g., tongue-touches), this would not have been effectively captured. However, it would be arguably misleading to call this instantaneous sampling, as the period between our “sample points” was only 2 seconds, which is much shorter than the period between sample points in typical instantaneous sampling methods (which is at least several minutes).

Note that we have provided our raw data with this manuscript and examining the spreadsheet “BehavData – NO MODIFIERS.csv” provides a comprehensive list of every behaviour that was measured, as well as the frequency and duration of each behaviour for each lizard at each period of observation. However, for the purposes of this manuscript, we have elected to focus only on the behaviours defined within the manuscript.

To make it clearer how behaviours were measured, we have added an extra sentence to the manuscript (new text underlined below):

“The analysis of videos to measure behaviour was completed using the BORIS software (Friard & Gamba, 2016). At each period of observation, the lizard’s behaviours were scored continuously. Definitions and additional details about the behaviours scored are provided below.”

Line 254: Why was the head used instead of the body? For example, if a pogona had its whole body in quadrant a and its head in quadrant b, was it considered to be in quadrant b?

Yes, the lizard would be considered to be in quadrant b in the situation described.

The lizard’s head was used to decide their location rather than their body for several reasons. Primarily, the head was considered to indicate the area the lizard “intended” to be in (although we do recognize that assuming the lizard’s intention can be problematic). The reason the head was considered to be the “intended” location was that the lizard’s head would either precede or follow the lizard’s movement. For example, if a lizard was walking towards the cork bark in the back left quadrant but paused right before walking onto the cork bark (i.e., no limbs would be touching the cork bark but their head would be “floating” over top), their body would be in the front left quadrant, but their head would be in the back left quadrant and would typically be facing the back wall. When movement resumed, lizards would almost always walk in the direction their head was facing, or, at the very least, would move almost fully into the quadrant their head was facing before turning around and moving into a different quadrant (lizards rarely backed up and were not really capable of tight turns). Therefore, it seemed more reasonable to assume that the lizard “intended” to be in the back left quadrant (or interacting with the cork bark), at least until their movement changed directions.

Furthermore, when lizards were adults, many had very large bodies, or could manipulate their torsos such that it would take up almost an entire quadrant. Therefore, if the torso was used to decide the lizard’s location, it may have appeared as though the lizards never exited certain quadrants, even when their front limbs, head, and a portion of their torso (i.e., up to the “collar bone”) were in a different location (or even interacting with different furnishings). It was also much easier to decide when the head was in a certain area, as the head could be clearly delineated from the rest of the body.

Finally, when the lizards hid or were in the camera’s blind spot, it was more likely that their head would be visible than their torso; lizards would often have their head poking out from under their hiding place or would sit with their body against the camera stand, leaving only their head in view. Therefore, even though their torso *may* have been in the same location as their head, we wanted to refrain from assuming too much about the lizard’s location in the enclosure.

We have added a sentence briefly justifying this choice in the methods (new text underlined below):

Lizards were considered to have crossed into a quadrant when the majority of their head was within that quadrant. The head was chosen as it could be easily delineated from the rest of the body and lizards almost always moved in the direction their head was pointing; therefore, this location was considered to represent their “intended” location.

Fig. 2 caption: Enter the information about the water basin, which was located in two quadrants.

Thank you for highlighting this omission. We have made the requested edit (new text underlined below):

A tile and basking lamp are present in the back right quadrant, a food bowl is present in the front right quadrant, a water bowl spans the front left and right quadrants, a paper hide is present in the front left quadrant, and the back left quadrant is empty.

Line 592: change Sample size for sample size

We have made the requested edit.

Lines 639-640: Did the rearing conditions vary between individuals? If so, how? If not, explain what you mean here.

Yes, the rearing conditions did differ between the two groups. Lizards were housed first in either naturalistic- or standard-style enclosures and were swapped into the opposite enclosure style closer to when they reached maturity (i.e., 1 year old). Therefore, some lizards were reared in standard-style enclosures, and others were reared in naturalistic-style enclosures. Note that this article is part of a larger body of work (the lead author’s PhD thesis) and the exact timing of rearing conditions will be discussed in future publications. However, rearing conditions are discussed in Table 1 and in the first paragraph under the heading “Enclosure styles”.

To make this clearer, we have added a small sentence in the area highlighted by the reviewer (new text underlined below):

However, considering that enclosure style failed to influence inactivity at all other time points, this could also be evidence that rearing conditions influence how *P. vitticeps* respond to novelty (half of the lizards lived in standard-style enclosures until enclosure styles were swapped at 1 year of age; see section titled “Enclosure styles” in methods and Table 1).

Lines 641-642: How enrichment influence these cited characteristics? Explain more and give examples.

We have added a sentence providing some more details about how enrichment can influence the characteristics cited. Note that, upon review, we also added some references to the original sentence.

New text (and any new references) underlined below:

Indeed, it is well-known that enrichment can influence neophobia, anxiety, and cognition in a variety of vertebrates (Gatto et al., 2022; Simpson & Kelly, 2011; Zentall, 2021) including snakes (Almli & Burghardt, 2006; Nagabaskaran et al., 2021, 2025). For example, as has been seen in rats (Simpson & Kelly, 2011), exposure to enrichment can increase the size certain brain regions (Nagabaskaran et al., 2025), reduce habituation time (Almli & Burghardt, 2006), and may improve learning or reduce neophobia (Nagabaskaran et al., 2021) in snakes. Regardless, either interpretation…

Lines 740-745: Most of the parameters analyzed did not differ between the two types of environment, but those that did resulted in better living conditions for the animals in the naturalistic enclosures. Wouldn't this in itself support the idea that naturalistic enclosures would be better, even without changing several parameters?

We appreciate this observation. Yes, the lines highlighted by the reviewer discuss how naturalistic enclosures offered better thermal heterogeneity and that female lizards in naturalistic enclosures had lower H:L ratios. While this does suggest that the naturalistic enclosures were somewhat better at accommodating the lizard’s behaviour or improving their welfare, the absence of actual behavioural evidence advise caution.

Superior thermal heterogeneity should improve the lizard’s ability to thermoregulate, but the absence of differences in their enclosure use and time spent inactive (both of which would provide insight about basking behaviour) highlights that this thermal heterogeneity may not actually be appreciated, as lizards do not appear to be actually *using* the thermal heterogeneity to its full effect. Of course, lizards do not have to “use” the thermal heterogeneity for it to be beneficial, but the absence of differences in relaxation-related postures (inactive and leg stretched, ILS) or stress-related behaviours (interacting with barriers, IWB) also suggests that there are no obvious improvements in the lizard’s welfare. Therefore, while the superior thermal heterogeneity may be considered an improvement independently of the behavioural data, this improvement is, evidently, not having a meaningful impact on the lizards.

Similarly, although the H:L ratios were higher for female lizards in standard enclosures, the relatively small sample size supporting this effect must suggest the interpretation that our results could be unrelated to enclosure size (as mentioned in the discussion for this result; lines 724-744). Furthermore, this relatively small effect also suggests that stressors unrelated to the enclosure size *per se* could have contributed to this result (e.g., the frequency of cage changes as mentioned in the discussion). Indeed, because physiological measurements of welfare such as H:L ratios can be non-specific to affective states (Davis et al., 2008; Mason & Mendl, 1993), this finding would have been bolstered if behavioural evidence had corroborated that female lizards in standard enclosures were experiencing higher stress. However, as mentioned before, the absence of behavioural evidence demonstrates that this result should be interpreted with caution.

**References cited in response:**

Davis, A. K., Maney, D. L., & Maerz, J. C. (2008). The use of leukocyte profiles to measure stress in vertebrates: A review for ecologists. Functional Ecology, 22(5), 760–772. https://doi.org/10.1111/j.1365-2435.2008.01467.x

Mason, G. J., & Mendl, M. (1993). Why is there no simple way of measuring animal welfare? Animal Welfare, 2, 301–319.

Lines 747-752: That was my main concern with the study. Was the so-called naturalistic enclosure really naturalistic? I didn't see it that way either. I really enjoyed seeing this paragraph here. My suggestion is that you post ideas on how to better structure an enclosure to the point where it is truly naturalistic. For example, if the animal is semi-arboreal, why aren't there structures like this in the enclosures?

We appreciate the reviewer’s kind words; we always strive to interpret our results in an objective, unbiased manner.

It is difficult to know *a priori* what modifications must be made so that the enclosure is “truly naturalistic”; indeed, this is somewhat discussed in the introduction of this manuscript (e.g., lines 72-78). We knew that *P. vitticeps* were semi-arboreal, and therefore we provided cork bark to accommodate the lizard’s potential need to climb. However, as mentioned in the discussion (e.g., lines 774-778), it is possible that the enclosures were too short (or the cork bark was too “low”) to effectively accommodate such behaviours. Furthermore, because we were also aware that *P. vitticeps* will dig or take advantage of rudimentary burrows (de Vosjoli et al., 2017; Doneley, 2006; Raiti, 2012; Read, 1995; Stahl, 1999), lizards were provided loose substrate. However, research in other species has shown that the design of the burrow may impact its use and therefore could impact its value (Ebrahimi et al., 2012). Similarly, research in gerbils has demonstrated that loose substrate does not ameliorate stereotypic digging, but the provision of a burrow does, suggesting that the need to hide in the substrate was important rather than the need to dig (Wiedenmayer, 1997). Therefore, while we may have some ideas as to what aspects of the natural habitat are important to the animals, which features of the natural habitat can actually improve (or are able to influence) animal welfare are more elusive.

For these reasons, many researchers have suggested that enclosure design should focus on accommodating important behaviours rather than effectively replicating what is “natural” (Dawkins, 1990; Veasey et al., 1996). Of course, the natural habitat of an animal is a highly effective starting place, but it should not be the sole focus – otherwise, enclosures may be aesthetically pleasing but not effective at improving an animal’s welfare (Fàbregas et al., 2012; Veasey et al., 1996). This is why we have interpreted some of our results in the context of accommodating particular behaviours (e.g., lines 791 – 804), and why our hypotheses focused on facilitating the lizard’s agency.

**References cited in response:**

Dawkins, M. S. (1990). From an animal’s point of view: Motivation, fitness, and animal welfare. *Behavioral and Brain Sciences*, *13*, 1–61.

de Vosjoli, P., Sommella, T. M., Mailloux, R., Donoghue, S., Klingenberg, R., & Cole, J. (2017). *The Bearded Dragon Manual: Expert Advice for Keeping and Caring for a Healthy Bearded Dragon.* (2nd ed.). Fox Chapel Publishers International Ltd.

Doneley, B. (2006). CARING FOR THE BEARDED DRAGON. *Proceedings of the North American Veterinary Conference*, *20*, 1607–1611.

Ebrahimi, M., Fenner, A. L., & Bull, C. M. (2012). Lizard behaviour suggests a new design for artificial burrows. *Wildlife Research*, *39*(4), 295. <https://doi.org/10.1071/WR11155>

Fàbregas, M. C., Guillén-Salazar, F., & Garcés-Narro, C. (2012). Do naturalistic enclosures provide suitable environments for zoo animals? *Zoo Biology*, *31*(3), 362–373. <https://doi.org/10.1002/zoo.20404>

Raiti, P. (2012). Husbandry, Diseases, and Veterinary Care of the Bearded Dragon (*Pogona vitticeps*). *Journal of Herpetological Medicine and Surgery*, *22*(3), 117–131. <https://doi.org/10.5818/1529-9651-22.3.117>

Stahl, S. J. (1999). General Husbandry and Captive Propagation of Bearded Dragons, *Pogona vitticeps*. *Bulletin of the Association of Reptilian and Amphibian Veterinarians*, *9*(4), 12–17. <https://doi.org/10.5818/1076-3139.9.4.12>

Veasey, J. S., Waran, N. K., & Young, R. J. (1996). ON COMPARING THE BEHAVIOUR OF ZOO HOUSED ANIMALS WITH WILD CONSPECIFICS AS A WELFARE INDICATOR. *Animal Welfare*, *5*, 13–24.

Wiedenmayer, C. (1997). Causation of the ontogenetic development of stereotypic digging in gerbils. *Animal Behaviour*, *53*(3), 461–470. <https://doi.org/10.1006/anbe.1996.0296>

**Reviewer #2:** This study investigates how enclosure design influences the behavior and welfare of bearded dragons (Pogona vitticeps) in artificial environments. The research compares naturalistic (complex) enclosures with standard (simple) enclosures to determine whether more complex environments improve the welfare of these lizards.

The manuscript is well written, well supported by literature, and presents highly relevant data for the management of Pogona vitticeps. However, one point draws my attention, and I would like more detailed clarification.

- How was the 28-day interval defined for blood collection in establishing the H:L ratio? What reference was used to establish this interval? If the response to environmental changes occurred acutely within a shorter time frame, the authors missed this important moment. Could the authors discuss the sensitivity of the H:L ratio in Pogona vitticeps and the possible implications of an inadequate collection interval, especially for the detection of acute changes?

The 28-day interval was simply the shortest time interval between blood samples; only 1 sample was actually taken within 28 days of another blood sample. Of the remaining 23 lizards, 10 were never re-sampled, 12 were re-sampled greater than 30 days after the previous sample, and 1 lizard was re-sampled greater than 60 days after their previous sample. The exact sensitivity of H:L ratios to acute stressors is not well-known in reptiles, and no research has examined this in *P. vitticeps* specifically. However, when corticosterone is artificially increased (i.e., by daily injections or surgical implants), H:L ratios may respond within 3-4 weeks (Davis et al., 2008).

In addition, as we collected H:L ratios to assess whether lizards experienced chronic stress in certain housing conditions, we did not intend for H:L ratios to capture the influence of any acute stressors. The 28-day interval was stated to demonstrate that the re-sampling of blood itself was unlikely to influence H:L ratios. Therefore, although taking blood samples likely caused short-term stress, this stressor was unlikely to be consistent or intense enough to result in marked changes in H:L ratios (as stated previously in this comment). Furthermore, only the actual drawing of blood would have been a novel experience; lizards were habituated to handling and mild restraint, as they had spent over 2 years being handled at least once per week prior to blood sampling, and the personnel who took blood samples were also familiar to the animals.

**References cited in response:**

Davis, A. K., Maney, D. L., & Maerz, J. C. (2008). The use of leukocyte profiles to measure stress in vertebrates: A review for ecologists. *Functional Ecology*, *22*(5), 760–772. <https://doi.org/10.1111/j.1365-2435.2008.01467.x>

- Another point that needs adjustment is the definition of a naturalistic enclosure. The modified enclosure cannot be considered naturalistic under any circumstances, as only a few physical structures and a substrate different from the initial phase were introduced.

We appreciate the concern. As discussed with the previous reviewer, we chose to label the “naturalistic” enclosure as naturalistic for several reasons. Primarily, “naturalistic” best described the enclosure’s appearance compared to the standard enclosures and also captured the intended outcome of the modifications in the naturalistic enclosure (i.e., to simulate aspects of the natural environment and promote natural behaviours). Although the naturalistic enclosures may also be described as “complex”, this label would have shifted the focus away from the rationale and intended outcome of the furnishings in the naturalistic enclosures. Indeed, because naturalistic enclosures included only 2 *additional* furnishings (cork bark with paper hide underneath) but *modified* 2 others (substrate and the naturalistic hide in the front-left quadrant), the term “complex” may have caused readers to overlook these modifications and focus on the number of furnishings.

Furthermore, the contrast between the standard and naturalistic enclosures may effectively replicate the conditions provided to pet reptiles in captivity. Reptiles are often provided, at minimum, a hide and a warm/basking spot (representing the “standard” enclosure), but loose substrate and climbing apparatuses may be less common depending on the species (Cargill et al., 2022; D’Cruze et al., 2020; Howell et al., 2022; Howell & Bennett, 2017). Therefore, our enclosure designs were chosen to have the broadest potential impact, as the pet trade represents a large portion of the reptiles currently kept in captivity (although *P. vitticeps* are also becoming an increasingly popular model organism for certain research; Fenk et al., 2024). Both enclosure designs were also approved by our local Animal Care Committee and represent aspects that may be feasible in a research environment.

The label “naturalistic” was also chosen as the label “enriched” may have been misleading. In the literature, “enriched” will typically (and should exclusively; Olsson & Dahlborn, 2002) refer to modifications that *improve* the animal’s welfare in some way, shape, or form (Ratuski & Weary, 2022). As we had no evidence that any of the resources provided to lizards improved the lizard’s well-being beyond the basic requirements, the label “enriched” was avoided. This is also why, throughout the article, we referred to the items in the enclosures as “resources” or “furnishings” rather than “enrichments” (a similar convention has been used in other literature; e.g., Cait et al., 2024).

Therefore, although we agree with the reviewer that the naturalistic enclosures did not closely replicate the lizard’s natural environment, these enclosures can still be viewed as “naturalistic” when compared to the standard enclosures. And, as this manuscript focused only on comparisons between these two enclosure styles, the label “naturalistic” was arguably the most accurate in the context of our experiment. Regardless, to make it clearer that we did not intend for “naturalistic” enclosures to fully replicate the natural environment, we have modified the following sentence in the introduction (new text underlined below):

Because naturalistic enclosures will include additional furnishings and attempt to mimic aspects of the natural environment, we expect these enclosures will better facilitate a lizard’s agency and that the welfare of lizards in these enclosures will be better than those in standard enclosures; we also expect this influence to be evident regardless of the order in which enclosure styles are experienced, and that an enclosure style’s influence will increase the longer it is inhabited.

**References cited in response**:

Cait, J., Winder, C. B., & Mason, G. J. (2024). How much “enrichment” is enough for laboratory rodents? A systematic review and meta-analysis re-assessing the impact of well-resourced cages on morbidity and mortality. *Applied Animal Behaviour Science*, *278*, 106361. <https://doi.org/10.1016/j.applanim.2024.106361>

Cargill, B., Benato, L., & Rooney, N. (2022). A survey exploring the impact of housing and husbandry on pet snake welfare. *Animal Welfare*, *31*(2), 193–208. <https://doi.org/10.7120/09627286.31.2.004>

D’Cruze, N., Paterson, S., Green, J., Megson, D., Warwick, C., Coulthard, E., Norrey, J., Auliya, M., & Carder, G. (2020). Dropping the Ball? The Welfare of Ball Pythons Traded in the EU and North America. *Animals*, *10*(3), 413. <https://doi.org/10.3390/ani10030413>

Howell, T. J., & Bennett, P. C. (2017). Despite their best efforts, pet lizard owners in Victoria, Australia, are not fully compliant with lizard care guidelines and may not meet all lizard welfare needs. *Journal of Veterinary Behavior*, *21*, 26–37. <https://doi.org/10.1016/j.jveb.2017.07.005>

Howell, T. J., Warwick, C., & Bennett, P. (2022). Pet management practices of frog and turtle owners in Victoria, Australia. *Veterinary Record*, *191*(12), e2180. <https://doi.org/10.1002/vetr.2180>

Olsson, I. A. S., & Dahlborn, K. (2002). Improving housing conditions for laboratory mice: A review of “environmental enrichment.” *Laboratory Animals*, *36*(3), 243–270. <https://doi.org/10.1258/002367702320162379>

Ratuski, A. S., & Weary, D. M. (2022). Environmental Enrichment for Rats and Mice Housed in Laboratories: A Metareview. *Animals*, *12*(4), Article 4. <https://doi.org/10.3390/ani12040414>
