# Supplementary material for: Influence of enclosure design on the behaviour and welfare of Pogona vitticeps
Source: PLoS One. 2025 Jun 5;20(6):e0322682. doi: 10.1371/journal.pone.0322682 (PMC12140227; doi:10.1371/journal.pone.0322682)
Supplement: Attachment — Submitted filename: Response_to_Reviewers_auresp_2.docx [file pone.0322682.s005.docx]

**General comments:**

Note that, since the manuscript was submitted originally, we have added some new references to the references section. Specifically, we added:

Reference #39: Wild KH, Roe JH, Schwanz L, Georges A, Sarre SD. Evolutionary stability inferred for a free ranging lizard with sex‐reversal. Mol Ecol 2022;31:2281–92. <https://doi.org/10.1111/mec.16404>.

Reference #46: Fenk LA, Baier F, Laurent G. The bearded dragon *Pogona vitticeps*. Nat Methods 2024;21:1964–6. <https://doi.org/10.1038/s41592-024-02485-2>.

Reference #47: Doneley B. CARING FOR THE BEARDED DRAGON. Proceedings of the North American Veterinary Conference, vol. 20, Orlando, Florida, USA: 2006, p. 1607–11.

Reference #49: Read JL. Subhabitat variability: A key to the high reptile diversity in chenopod shrublands. Aust J Ecol 1995;20:494–501. <https://doi.org/10.1111/j.1442-9993.1995.tb00568.x>.

Reference #50: Stahl SJ. General Husbandry and Captive Propagation of Bearded Dragons, *Pogona vitticeps.* Bulletin of the Association of Reptilian and Amphibian Veterinarians 1999;9:12–7. <https://doi.org/10.5818/1076-3139.9.4.12>.

Reference #51: de Vosjoli P, Sommella TM, Mailloux R, Donoghue S, Klingenberg R, Cole J. The Bearded Dragon Manual: Expert Advice for Keeping and Caring for a Healthy Bearded Dragon. 2nd ed. West Sussex, U.K.: Fox Chapel Publishers International Ltd; 2017.

Reference #53: Olsson IAS, Dahlborn K. Improving housing conditions for laboratory mice: a review of “environmental enrichment.” Lab Anim 2002;36:243–70. <https://doi.org/10.1258/002367702320162379>.

Reference #54: Mendyk RW, Augustine L. Controlled Deprivation and Enrichment. In: Warwick C, Arena PC, Burghardt GM, editors. Health and Welfare of Captive Reptiles, Cham: Springer International Publishing; 2023. <https://doi.org/10.1007/978-3-030-86012-7>.

Reference #55: Ratuski AS, Weary DM. Environmental Enrichment for Rats and Mice Housed in Laboratories: A Metareview. Animals 2022;12:414. <https://doi.org/10.3390/ani12040414>.

Reference #56: Cait J, Winder CB, Mason GJ. How much “enrichment” is enough for laboratory rodents? A systematic review and meta-analysis re-assessing the impact of well-resourced cages on morbidity and mortality. Appl Anim Behav Sci 2024;278:106361. <https://doi.org/10.1016/j.applanim.2024.106361>.

Reference #57: Williams DL, Jackson R. Availability of Information on Reptile Health and Welfare from Stores Selling Reptiles. Open J Vet Med 2016;06:59–67. <https://doi.org/10.4236/ojvm.2016.63007>.

Reference #58: Cargill B, Benato L, Rooney N. A survey exploring the impact of housing and husbandry on pet snake welfare. Anim Welf 2022;31:193–208. <https://doi.org/10.7120/09627286.31.2.004>.

Reference #59: D’Cruze N, Paterson S, Green J, Megson D, Warwick C, Coulthard E, et al. Dropping the Ball? The Welfare of Ball Pythons Traded in the EU and North America. Animals 2020;10:413. <https://doi.org/10.3390/ani10030413>.

Reference #60: Howell TJ, Bennett PC. Despite their best efforts, pet lizard owners in Victoria, Australia, are not fully compliant with lizard care guidelines and may not meet all lizard welfare needs. J Vet Behav 2017;21:26–37. <https://doi.org/10.1016/j.jveb.2017.07.005>.

Reference #61: Howell TJ, Warwick C, Bennett P. Pet management practices of frog and turtle owners in Victoria, Australia. Vet Rec 2022;191:e2180. <https://doi.org/10.1002/vetr.2180>.

Reference #84: Simpson J, Kelly JP. The impact of environmental enrichment in laboratory rats—Behavioural and neurochemical aspects. Behav Brain Res 2011;222:246–64. https://doi.org/10.1016/j.bbr.2011.04.002.

Reference #85: Gatto E, Dadda M, Bruzzone M, Chiarello E, De Russi G, Maschio MD, et al. Environmental enrichment decreases anxiety‐like behavior in zebrafish larvae. Dev Psychobiol 2022;64:e22255. <https://doi.org/10.1002/dev.22255>.

Reference #87: Nagabaskaran G, Moonilal V, Skinner M, Miller N. Environmental Enrichment Increases Brain Volume in Snakes. J Comp Neurol 2025;533:e70043. <https://doi.org/10.1002/cne.70043>.

In addition, reference #73 in the original manuscript has been moved to reference #48:

Reference #48: MacMillen RE, Augee ML, Ellis BA. Thermal ecology and diet of some xerophilous lizards from western New South Wales. J Arid Environ 1989;16:193–201. <https://doi.org/10.1016/S0140-1963(18)31026-7>.

**Responses to reviewers:**

Reviewer #1: Dear authors, thank you very much for resubmitting your work.
Congratulations on the revision of the manuscript and thank you for considering my suggestions. I was very pleased with the responses, I agree with them and I approve the changes made to the body of the paper. I believe that the work is clearer for readers. However, I suggest three more small changes to the manuscript:
1 - In the abstract, on lines 21 and 22, I suggest changing “perfectly replicate native” to “that intend to replicate native”. This is based on your answers to the previous questions. As it stands, it looks like you did this in the study, which was not the case. If you don't think this suggestion is ideal, then I suggest you just rewrite this sentence and the next one so that it doesn't look like you're testing a perfectly naturalistic enclosure.

Thank you for highlighting how this phrasing could be misleading. We have made the requested edit, although we chose slightly different wording (new text underlined below):

However, designing such enclosures can be difficult if little is known about the animal in the wild, and enclosures that aim to replicate natural habitats must still be assessed to ensure their assumed benefits are realized.

2 - In line 573, replace & with and, just to keep the text standard.

We have made the requested edit.

3 - The explanation of why the term naturalistic was used in the reply letter was excellent. My suggestion is to insert it in the manuscript. I suggest you do this in the Enclosure style methodology section. This explanation really clarifies the researchers' thinking and the justification is quite acceptable.

We appreciate the comment. As suggested, we have added 2 paragraphs within the methods justifying our enclosure designs and terminology. These paragraphs appear immediately after the figure 1 caption. These paragraphs heavily resemble our original response, but we have edited them to improve the flow of ideas and remove improper phrasing (i.e., references to “the readers”).

Well, those are my final suggestions. Once again, I congratulate the authors on their excellent work.

Thank you for your revisions as well! We have appreciated your professional tone and insightful comments.
